# Whole-brain mapping of the direct inputs and axonal projections of POMC and AgRP neurons

**DOI:** 10.3389/fnana.2015.00040

**Published:** 2015-03-27

**Authors:** Daqing Wang, Xiaobing He, Zhe Zhao, Qiru Feng, Rui Lin, Yue Sun, Ting Ding, Fuqiang Xu, Minmin Luo, Cheng Zhan

**Affiliations:** ^1^School of Life Sciences, Tsinghua UniversityBeijing China; ^2^National Institute of Biological SciencesBeijing, China; ^3^Key Laboratory of Magnetic Resonance in Biological Systems and State Key Laboratory of Magnetic Resonance and Atomic and Molecular Physics, Wuhan Institute of Physics and Mathematics, Chinese Academy of SciencesWuhan, China; ^4^University of Chinese Academy of SciencesBeijing, China; ^5^Wuhan National Laboratory for OptoelectronicsWuhan, China

**Keywords:** arcuate nucleus, POMC neurons, nucleus tractus solitarius, transsynaptic tracing, rabies virus, adeno-associated virus

## Abstract

Pro-opiomelanocortin (POMC) neurons in the arcuate nucleus (ARC) of the hypothalamus and nucleus tractus solitarius (NTS) of the brainstem play important roles in suppressing food intake and maintaining energy homeostasis. Previous tract-tracing studies have revealed the axonal connection patterns of these two brain areas, but the intermingling of POMC neurons with other neuron types has made it challenging to precisely identify the inputs and outputs of POMC neurons. In this study, we used the modified rabies virus to map the brain areas that provide direct inputs to the POMC neurons in the ARC and NTS as well as the inputs to the ARC AgRP neurons for comparison. ARC POMC neurons receive inputs from dozens of discrete structures throughout the forebrain and brainstem. The brain areas containing the presynaptic partners of ARC POMC neurons largely overlap with those of ARC AgRP neurons, although POMC neurons receive relatively broader, denser inputs. Furthermore, POMC neurons in the NTS receive direct inputs predominantly from the brainstem and show very different innervation patterns for POMC neurons in the ARC. By selectively expressing fluorescent markers in the ARC and NTS POMC neurons, we found that almost all of their major presynaptic partners are innervated by POMC neurons in the two areas, suggesting that there are strong reciprocal projections among the major POMC neural pathways. By comprehensively chartering the whole-brain connections of the central melanocortin system in a cell-type-specific manner, this study lays the foundation for dissecting the roles and underlying circuit mechanisms of specific neural pathways in regulating energy homeostasis.

## Introduction

The central melanocortin system plays important roles in regulating energy homeostasis, cardiovascular function, and reproduction (Cone, 2005; Morton et al., 2006; Millington, 2007). This system includes three groups of projection neurons (Cone, 2005): POMC neurons in the arcuate nucleus (ARC) of the hypothalamus, POMC neurons in the nucleus tractus solitarius (NTS) of the medulla, and AgRP neurons in the ARC. Studies in previous decades have revealed apparently opposite functions for POMC neurons and AgRP neurons in regulating food intake. Stimulating POMC neurons in the ARC and NTS suppresses feeding (Aponte et al., 2011; Zhan et al., 2013), whereas stimulating AgRP neurons rapidly elicits feeding behavior (Aponte et al., 2011; Krashes et al., 2011). By contrast, killing POMC neurons induces hyperphagia and obesity (Yaswen et al., 1999; Coll et al., 2004; Xu et al., 2005; Zhan et al., 2013), whereas ablating AgRP neurons in adult mice induces hypophagia and, ultimately, starvation (Gropp et al., 2005; Luquet et al., 2005).

Classic tract tracings have revealed that neurons in the ARC and NTS receive inputs from, and project to, broad brain areas (Makara and Hodacs, 1975; Ricardo and Koh, 1978; Schwaber et al., 1982; Chronwall, 1985; Gruber et al., 1987; Sim and Joseph, 1991; Magoul et al., 1993; Rinaman, 2010). Different cell types are intermingled in both brain areas, thus making it impossible to assign conventional tracing results to specific types of neurons among nuclei. The recent development of the rabies virus-based transynaptic tracing technique has enabled the precise mapping of direct inputs to various cell populations in a cell-type specific manner (Wickersham et al., 2007; Wall et al., 2010, 2013; Osakada et al., 2011; Arenkiel et al., 2012; Garcia et al., 2012; Watabe-Uchida et al., 2012; Ogawa et al., 2014; Pollak Dorocic et al., 2014; Stanek et al., 2014; Weissbourd et al., 2014). Using this strategy, Krashes and colleagues have unraveled the pattern of presynaptic inputs to AgRP neurons in the ARC (Krashes et al., 2014). However, the whole-brain inputs to POMC neurons have not yet been examined. In view of the outputs, the axon projection patterns of the AgRP neurons have been studied using the AAV-based anterograde tracing technique (Atasoy et al., 2008; Betley et al., 2013), but the outputs of POMC neurons in the ARC and NTS have not been systemically studied.

Here we used the modified rabies virus and the Cre/loxp gene expression system to map the whole-brain distribution patterns of the neurons that provide direct inputs to POMC neurons in the ARC and NTS. In addition, we studied the axon projection patterns of these two sets of POMC neurons by separately expressing fluorescent proteins of different colors. The input and output profiles of POMC neurons were compared to those of ARC AgRP neurons. Our results reveal distinct connectivity between POMC neurons in the ARC and NTS. Moreover, we show that POMC neurons and AgRP neurons form rich reciprocal connections with their respective upstream stations. The comprehensive mapping of connection patterns outlines the structural framework for future systematic studies of the neural circuits that underlie the behavioral and endocrinological functions of brain POMC neurons.

## Materials and methods

### Mice

The animal care and use conformed to the institutional guidelines of National Institute of Biological Sciences, Beijing and the governmental regulations of China. Mice were housed under a controlled temperature (22–25°C) and 12 h light-dark cycle with standard mouse chow and water provided *ad libitum*. We used adult mice (2–4 month) of either sex. POMC-Cre [Jackson Laboratory strain name Tg(Pomc1-cre)16Lowl/J] (Balthasar et al., 2004) and AgRP-Cre [Jackson Laboratory strain Name Agrptm1(cre)Lowl/J] (Tong et al., 2008) mouse lines were backcrossed and maintained in a C57BL6 background. Littermate wildtype mice were used for control experiments.

### Viral vectors production

The initial rabies viruses SAD-ΔG-mCherry (EnvA) and the cell lines for rabies propagation and titering were kindly sied by E.M. Callaway at Salt Institute. The rabies viruses were produced and concentrated as previously described (Osakada et al., 2011). The final titer of EnvA-RV-mCherry was 2 × 10^8^ infecting unit per milliliter.

Cre-dependent adeno-associated virus (AAV) plasmids with a DIO sequence carrying rabies glycoprotein (RG) or TVA were generated as helper virus vectors for retrograde transsynaptic tracing. The AAV-DIO-EGFP-TVA plasmid was constructed by sub-cloning the CAG promoter from AAV-CAG-GFP-ires-CRE plasmid (Addgene plasmid 48201) and the coding region of GFP:2A:TVA from the AAV-EF1a-FLEX-GT plasmid (Addgene plasmid 26198) (Wall et al., 2010) into the DIO cassette of the plasmid pAAV-EF1a-DIO-hChR2(H134R)-EYFP (Addgene plasmid 20298). The AAV-DIO-RG plasmid was constructed by sub-cloning the CAG promoter from the AAV-CAG-GFP-ires-CRE plasmid (Addgene plasmid 48201) and coding region of RG from AAV-EF1a-FLEX-GTB (Addgene plasmid 26197) (Haubensak et al., 2010) into the DIO cassette of the plasmid pAAV-EF1a-DIO-hChR2(H134R)-EYFP (Addgene plasmid 20298).

Another two Cre-dependent AAV plasmids were generated for tracing axonal projections. pAAV-EF1a-DIO-EmGFP and pAAV-EF1a-DIO-mtdTomato were constructed by replacing the coding region of hChR2(H134R)-EYFP with the coding sequence of the membrane-bound form of EGFP (EmGFP; Addgene plasmid 14757) (Matsuda and Cepko, 2007) or membrane-bound form of tdTomato in the AAV-EF1a-DIO-hChR2(H134R)-EYFP-WPRE-HGHpA plasmid (Addgene plasmid 20298). All AAV vectors were packaged into 2/9 serotypes with titers of approximately 2 × 10^12^ genome copies per milliliter.

### Stereotaxic virus injection

To perform stereotaxic viral injections, mice were anesthetized with pentobarbital (i.p. 80 mg/kg) and then mounted in a stereotaxic holder. A small incision was made in the skin to expose the skull. After thoroughly cleaning the skull with 0.3% hydrogen peroxide solution, we drilled a small hole through the skull for virus injection. For cell-type-specific retrograde tracing, two Cre-dependent AAV-DIO-EGFP-TVA and AAV-DIO-RG were mixed with an equal volume prior to viral injections; then, 80–300 nl AAV mixtures filled in a pulled glass pipettes were stereotaxically injected into target areas (ARC coordinate AP/DV/ML: -1.7/-5.5/-0.2 mm; NTS coordinate AP/DV/ML: 8.4/3.3/0 mm) of POMC-Cre or AgRP-Cre mice using a microsyringe pump (Nanoliter 2000 Injector, WPI), which allowed EGFP-TVA and RG selectively expression in POMC or AgRP neurons. After 3 weeks of recovery and AAV expression, 300 nl SAD19ΔG-mCherry(EnvA) was injected into the same location in a biosafety level-2 environment. After 1 week of rabies virus infection and transsynaptic spread (Wall et al., 2010; Watabe-Uchida et al., 2012), the animals were sacrificed. To directly compare the axonal projections of POMC neurons in the ARC vs. the NTS, 300 nl anterograde tracer AAV-DIO-mtdTomato or AAV-DIO-EmGFP was injected into the ARC or NTS respectively. Similarly, 300 nl AAV-DIO-EmGFP was injected into the ARC of AgRP-Cre mice for anterograde tracing. To achieve strong labeling of the axons, animals were allowed to survive for 1 month post surgery (Gautron et al., 2010).

### Histology and immunostaining

Mice were anesthetized by i.p. injection of an overdose of pentobarbital, and then transcardially perfused with 0.9% saline followed by 4% paraformaldehyde (PFA) in PBS. After post fixation overnight, the brain was isolated and cryoprotected with 30% sucrose for 2 days. For further imaging and analysis, whole brains were coated with tissue freezing medium and coronal sections (40 μm thick) were prepared on a cryostat (Leica CM1900). Some mouse brains were cut sagittally to better visualize the axon projections. Brain sections mounted on chrome-gelatin subbed slides were washed with PBS four times every 6 min. For immunofluorescent staining, the sections were blocked with 3% BSA in PBS-0.3% Triton X-100 and subsequently incubated with primary antibodies rabbit anti-POMC (1:200, catalog# H-029-30, Phoenix Pharmaceuticals; overnight), goat anti-AgRP (15 μg/ml, catalog# GT15023, Neuromics; 72 h), or chicken anti-GFP (1:500, catalog#ab290, Abcam; overnight) at 4°C. Sections were then incubated with secondary antibodies, Alexa647 donkey anti-rabbit (1:500, Jackson ImmunoResearch), Alexa647 donkey anti-goat (1:500, Jackson ImmunoResearch) or Cy2 anti-rabbit (1:500, Jackson ImmunoResearch) for 4 h at room temperature. Finally, these brain sections were cover-slipped with 50% DAPI-glycerol mounting medium.

### Imaging and data analysis

Whole brain sections were imaged with an automated slide scanner (VS120 Virtual Slide, Olympus) or a confocal microscope (DigitalEclipse A1, Nikon). The locations of the labeled neurons and outlines of the brain nuclei were manually defined in the commercial software Imaris (Bitplane, Zurich, Switzerland) according to the mouse brain atlas (Paxinos and Franklin, 2001). The cell number of each nucleus was counted automatically. Brightness, contrast and pseudocolor were adjusted, as needed, using ImageJ (NIH). For three-dimensional (3D) visualization, each imaged section was translated and rotated to align with its precursor image along the anteroposterior axis using AutoAligner (Bitplane). 3D visualizations of whole-brain inputs were generated with well-aligned image sequences using Imaris (Bitplane). For better visualization, each input neuron was represented as a red dot in the 3D models.

## Results

### Transynaptic labeling of direct inputs to POMC and AgRP neurons using rabies virus and Cre-loxP gene expression

**Figure 1:**
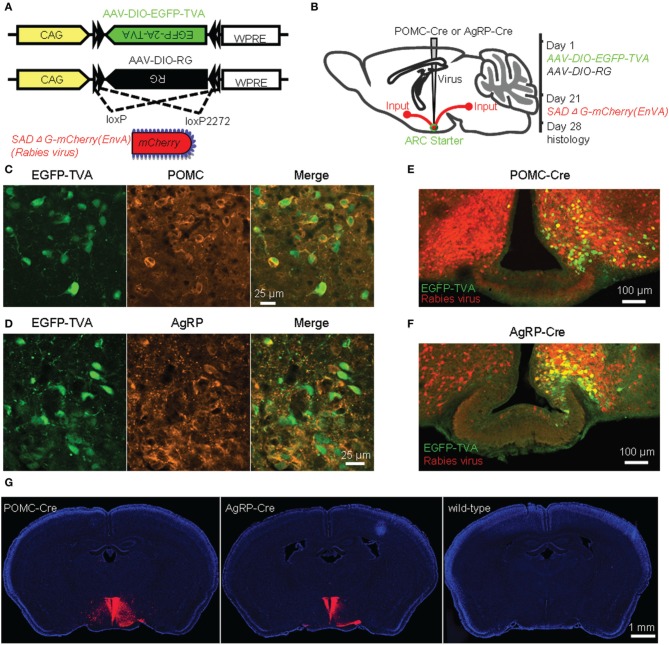
**Identification of direct inputs to POMC and AgRP neurons in the ARC using stereotaxic injection of the modified rabies virus into Cre-transgenic mice. (A)** Design of recombinant AAV-DIO-EGFP-TVA, AAV-DIO-RG, and modified rabies virus. **(B)** Experimental design. **(C)** Selective expression of EGFP-TVA in POMC-expressing neurons following the unilateral injection of AAV-DIO-EGFP-TVA into the ARC of a POMC-Cre mouse. **(D)**, EGFP-TVA expression pattern in the ARC of an AgRP-Cre mouse. **(E,F)** Characterization of the distribution pattern of starter neurons (green) and retrogradely labeled neurons (red) in the ARC of POMC-Cre **(E)** and AgRP-Cre **(F)** mice. **(G)** Transsynaptic labeling (red) was observed in a Cre-transgenic mouse but not a wildtype control mouse. Blue indicates cell nuclear staining with DAPI.

To achieve cell-type-specific retrograde transsynaptic tracing, we used the recently developed three-virus system in combination with the Cre/loxP gene-expression technique (Figure 1A) (Watabe-Uchida et al., 2012; Wall et al., 2013; Pollak Dorocic et al., 2014; Weissbourd et al., 2014). The modified rabies virus SAD-ΔG-mCherry(EnvA) was pseudotyped with the avian sarcoma leucosis virus envelope protein (EnvA), which allows the virus to selectively infect mammalian neurons that express TVA, the cognate receptor of EnvA. Additionally, the rabies glycoprotein (RG) gene required for transsynaptic spreading beyond initially infected neurons was replaced with the coding sequence of a red fluorescent protein, mCherry. Two Cre-dependent AAV recombinants, AAV-DIO-EGFP-TVA and AAV-DIO-RG, were stereotaxically infused into the unilateral ARC of POMC-Cre or AgRP-Cre mice (Figures 1A,B). After 3 weeks of the expression of EGFP-TVA and RG, the rabies virus SAD-ΔG-mCherry(EnvA) was injected into the same area. One week later, allowing for virus replication and transsynaptic spread, mouse brains were histologically prepared for examining the labeling patterns (Figure 1B).

EGFP-TVA signals were colocalized with POMC or AgRP immunoreactivity in the majority (~95%) of the neurons in the ARC (Figures 1C,D), confirming the accuracy of these two driver mouse lines (Balthasar et al., 2004; Tong et al., 2008). Starter neurons were characterized by the coexpression of RV-mCherry and EGFP-TVA, which was restricted within the ARC, unilateral to the injection site (Figures 1E,F). A substantial number of mCherry-positive neurons in the bilateral ARC did not express EGFP-TVA, suggesting the presence of local inputs (Figures 1E,F). We observed a large number of mCherry-expressing neurons outside of the ARC of POMC-Cre and AgRP-Cre mice (Figure 1G left and middle panels). In contrast, we did not detect any mCherry-positive neurons in wildtype littermates that were tested with the same procedures (Figure 1G right panel). These results demonstrated that the mCherry signals outside the injection sites are produced by the spread of rabies virus from Cre-expressing starter neurons.

### Input patterns of the ARC POMC and AgRP neurons

**Figure 2:**
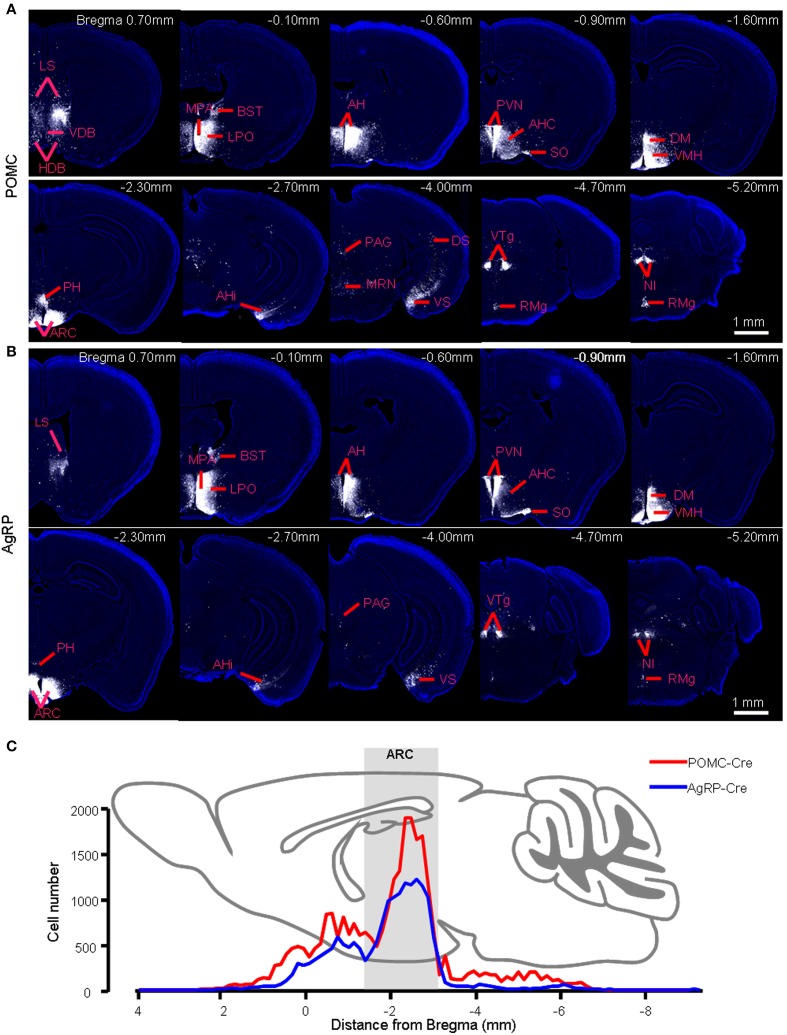
**Brain areas that provide presynaptic inputs to the ARC POMC and AgRP neurons. (A)** Series of coronal sections show the pattern of retrograde labeling following rabies virus injection into the right hemisphere of a POMC-Cre mouse. Rabies virus labeling is pseudocolored for better contrast against the DAPI-counterstaining of cell nuclei (blue). **(B)** The retrograde labeling pattern of an AgRP-Cre mouse. **(C)** Distribution profiles of input neuron numbers in each brain section of a POMC-Cre mouse and an AgRP-Cre mouse along the anteroposterior axis.

In POMC-Cre mice that were injected with the three viral vectors in the ARC, mCherry-labeled presynaptic neurons were located in dozens of discrete brain areas, including the lateral septum (LS), medial preoptic area and medial preoptic nucleus (MPA/MPO), anterior hypothalamus (AH), paraventricular hypothalamic nucleus (PVN), dorsomedial hypothalamus (DM), posterior hypothalamus (PH), amygdalohippocampal area (AHi), dorsal and ventral parts of subiculum (DS and VS), ventral tegmental nucleus (VTg), and nucleus incertus (NI) (Figure 2A). The presynaptic labeling was always bilateral, although it tended to be stronger in the hemisphere ipsilateral to the starter neurons. Despite the apparently opposite behavioral functions of POMC and AgRP neurons, the overall labeling pattern in POMC-Cre mice resembled that in AgRP-Cre mice at the level of brain nuclei (Figure 2B). 3D views of whole-brain inputs were constructed for better visualization (Supplementary movie 1 for a POMC-Cre mouse and Supplementary movie 2 for an AgRP-Cre mouse). Quantification of the number of labeled neurons in the individual coronal sections revealed a similar distribution along the anteroposterior axis, although ARC POMC neurons were innervated by more input neurons than ARC AgRP neurons (Figure 2C). On average, ARC POMC neurons received direct inputs from 43990 ± 8596 neurons (mean ± SEM) in the entire brain (*n* = 4 POMC-Cre mice). In contrast, ARC AgRP neurons received direct inputs from 17191 ± 4526 neurons (*n* = 5 AgRP-Cre mice). The numbers of starter cells in the ARC of these two mouse lines were similar (~900 for the POMC-Cre line vs. ~800 for the AgRP-Cre line), suggesting that the substantial difference in the number of input neurons reflects more inputs and a higher convergence ratio for POMC neurons (~49 for POMC vs. ~21 for AgRP).

**Figure 3:**
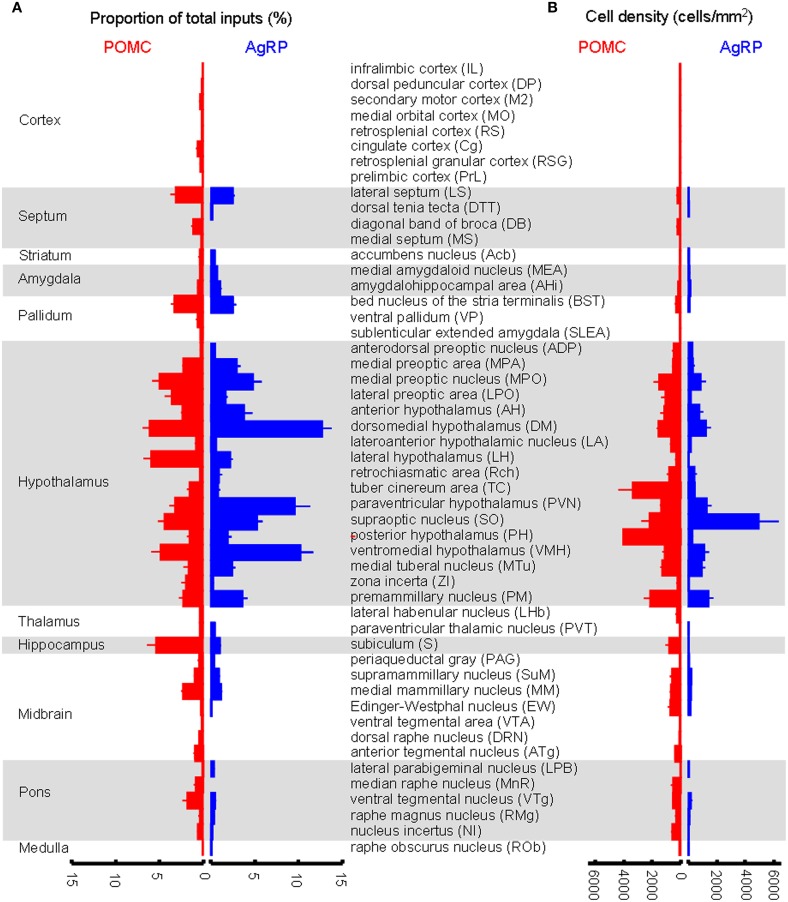
**Comparison of brain areas between POMC and AgRP neurons in the ARC. (A)** Proportion of input neurons for ARC POMC neurons (red) and AgRP neurons (blue). The values are the normalized ratio of the cell number in each area against the total number of input neurons. Error bars represent the SEM in this and the following figures. *n* = 4 POMC-Cre mice and 5 AgRP-Cre mice. **(B)** Cell density of input neurons in each brain area.

We further measured the number of labeled neurons and the labeling density in individual brain areas. The locations of labeled neurons were determined using a standard mouse atlas (Paxinos and Franklin, 2001). To minimize bias, only brain areas with at least 10 labeled neurons in at least one mouse line were analyzed. To correct potential bias, the cell number in each nucleus was further normalized by the total inputs. A list of whole brain inputs was generated for the ARC POMC and AgRP neurons (Figure 3). Overall, the hypothalamic areas provided the majority of inputs to both POMC (~60%) and AgRP (~70%) neurons in the ARC. The hypothalamic input areas mainly included the AH, DM, lateroanterior hypothalamic nucleus (LA), PVN, lateral hypothalamus (LH), supraoptic nucleus (SO), ventromedial hypothalamic nucleus (VMH), PH, MPA/MPO, and lateral preoptic area (LPO). Although the DM provided the largest number of inputs for POMC neurons, the SO was the most densely labeled area. The major forebrain input areas outside the hypothalamus include the subiculum (S) in the hippocampus, LS in the septum, and bed nucleus of the stria terminalis (BST) in the pallidum (~15% for POMC and ~10% for AgRP). No substantial labeling was found in a majority of the cortical areas, thalamus, or striatum. Beyond the forebrain, a few discrete nuclei in the midbrain and pons contained the remaining inputs. These areas mainly include the medial mammillary nucleus (MM), median raphe, and pontine central gray. Although the NTS is the major brainstem area for regulating energy homeostasis (Cone, 2005; Zhang et al., 2010; Wu et al., 2012; Young, 2012), we did not find any labeled neurons in this area for either POMC-Cre mice or AgRP-Cre mice.

**Figure 4:**
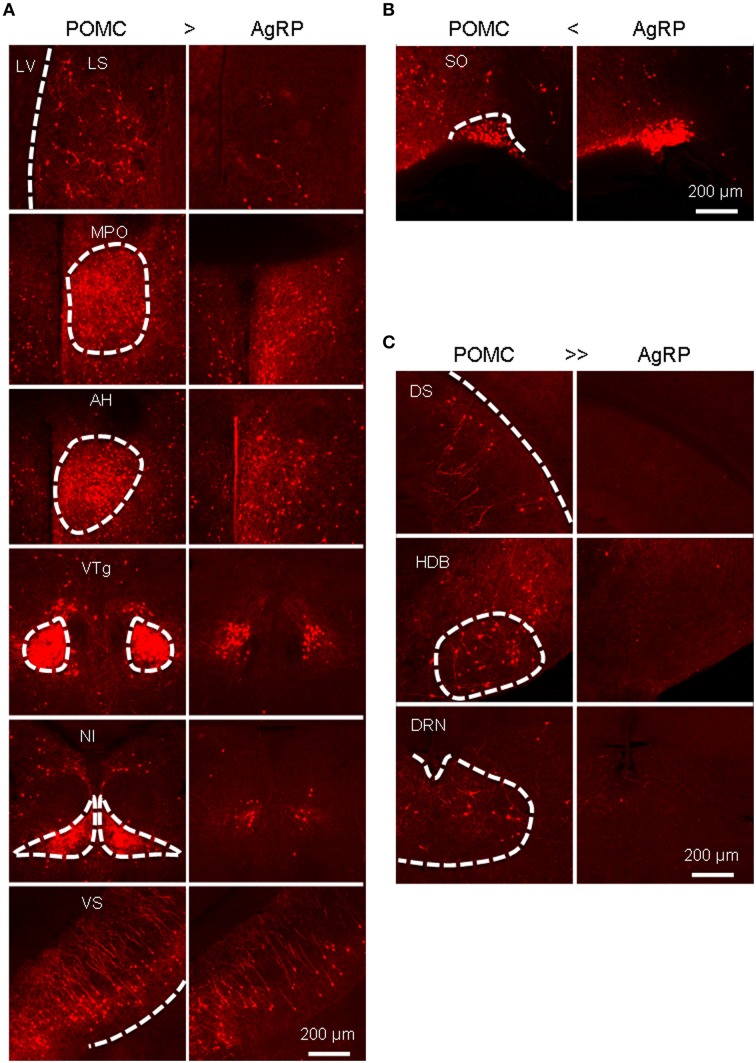
**Areas that provide different inputs to POMC and AgRP neurons in the ARC. (A)** Representative brain areas that provide stronger inputs to POMC neurons than to AgRP neurons. **(B)** The supraoptic nucleus (SO) sends more inputs to AgRP neurons than to POMC neurons. **(C)** Representative brain areas that solely target POMC neurons but not AgRP neurons.

A total of 52 brain areas provide direct inputs to the ARC POMC neurons, while 35 of the 52 areas also project to AgRP neurons. Within the 35 brain areas, the cell density of POMC-targeting neurons was often significantly higher than that of AgRP-targeting neurons (Figure 3). Figure 4A shows several such examples, including the LS, MPO, AH, VTg, NI, and VS. One exception was the SO, which was the most densely labeled nucleus in AgRP mice and had more input neurons for AgRP neurons than for POMC neurons (Figure 4B). The 17 brain areas selectively targeting ARC POMC neurons constituted only a small proportion (~7%) of the total inputs for POMC neurons. Figure 4C shows the labeling pattern of three such examples, including in the dorsal subiculum, horizontal diagonal band of broca (HDB), and dorsal raphe nucleus (DRN).

**Figure 5:**
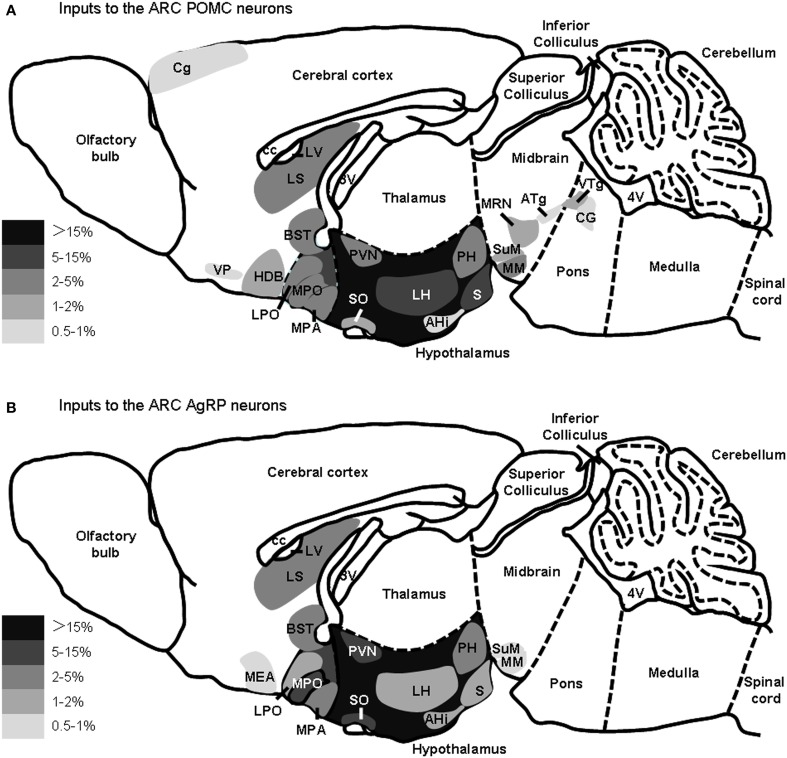
**Summary of the major presynaptic inputs to POMC neurons and AgRP neurons in the ARC**. Brain areas that make up greater than 0.5% of total inputs to POMC neurons **(A)** or AgRP **(B)** neurons in the ARC are shown on a schematic sagittal section, respectively. Gray scale represents the percentage of total inputs. LV, lateral ventricle; 3V, third ventricle; 4V, fourth ventricle.

The major presynaptic partners of POMC neurons and AgRP neurons in the ARC are summarized in Figure 5. Overall, the hypothalamic areas and several other forebrain nuclei represent the major input sources for both POMC and AgRP neurons in the ARC, while some nuclei in the midbrain and pons prefer to connect to ARC POMC neurons.

### Input patterns of NTS POMC neurons

In addition to the ARC, the NTS in the medulla also contains a substantial number of POMC-expressing neurons (Bronstein et al., 1992; Padilla et al., 2012). These NTS POMC neurons have been implicated in feeding behavior and energy metabolism (Rinaman et al., 1998; Fan et al., 2004; Zheng et al., 2005; Huo et al., 2006; Zhan et al., 2013). We asked whether the NTS POMC neurons receive direct inputs from the same sets of nuclei as those of the ARC POMC neurons.

**Figure 6:**
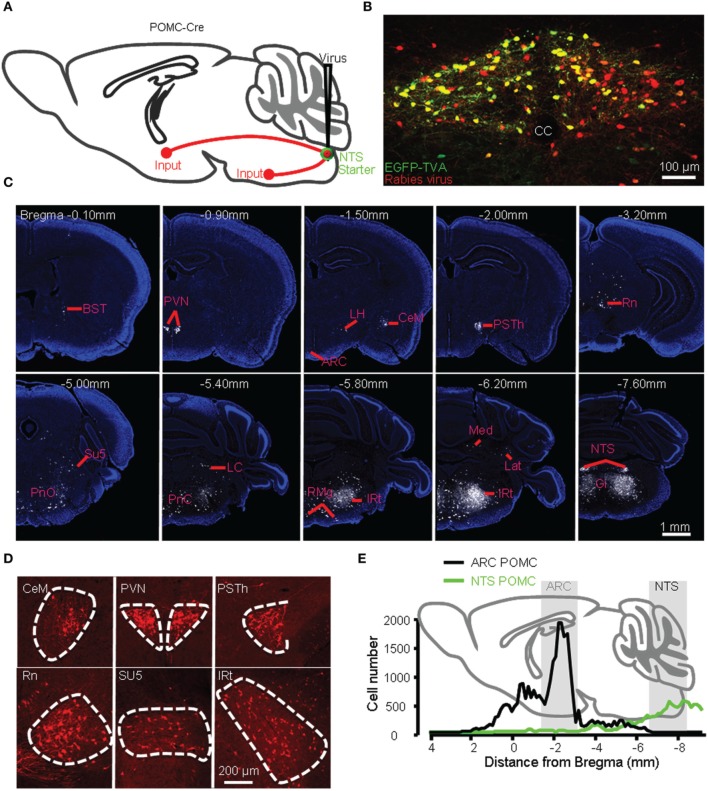
**Identification of presynaptic inputs to POMC neurons in the NTS. (A)** Modified rabies virus was injected into the NTS of POMC-Cre mice. **(B)** Starter neurons (yellow) were restricted within the NTS. cc, central canal. Representative coronal sections **(C)** and several densely labeled nuclei **(D)**. **(E)** Numbers of input neurons to POMC neurons in the ARC (black line) vs. NTS (green line) from anterior to posterior.

We injected AAV-DIO-EGFP-TVA and AAV-DIO-G viral vectors and then the modified rabies virus into the NTS of POMC-Cre mice (Figure 6A). The entire mouse brain was sectioned coronally, and both EGFP-TVA and mCherry signals were imaged for all brain sections. The starter neurons were present only in the NTS (Figure 6B). We observed retrogradely labeled, mCherry-expressing neurons mainly in the BST, PVN, LH, medial part of the central amygdaloid nucleus (CeM), parasubthalamic nucleus (PSTh), red nucleus (Rn), oral and caudal parts of the pontine reticular nucleus (PnO and PnC), locus coeruleus (LC), intermediate reticular nucleus (IRt), gigantocellular reticular nucleus (Gi), raphe magnus nucleus (RMg), lateral and medial cerebellar nucleus (Lat and Med) (Figures 6C,D). The 3D reconstruction of whole-brain inputs to the NTS POMC neurons was shown in Supplementary movie 3. Overall, the starter neurons in the NTS (~300) received direct inputs from 22,061 ± 5092 neurons (*n* = 5 POMC-Cre mice). Therefore, the convergence ratio for NTS POMC neurons is much higher than that for ARC POMC neurons (~74 vs. ~49).

**Figure 7:**
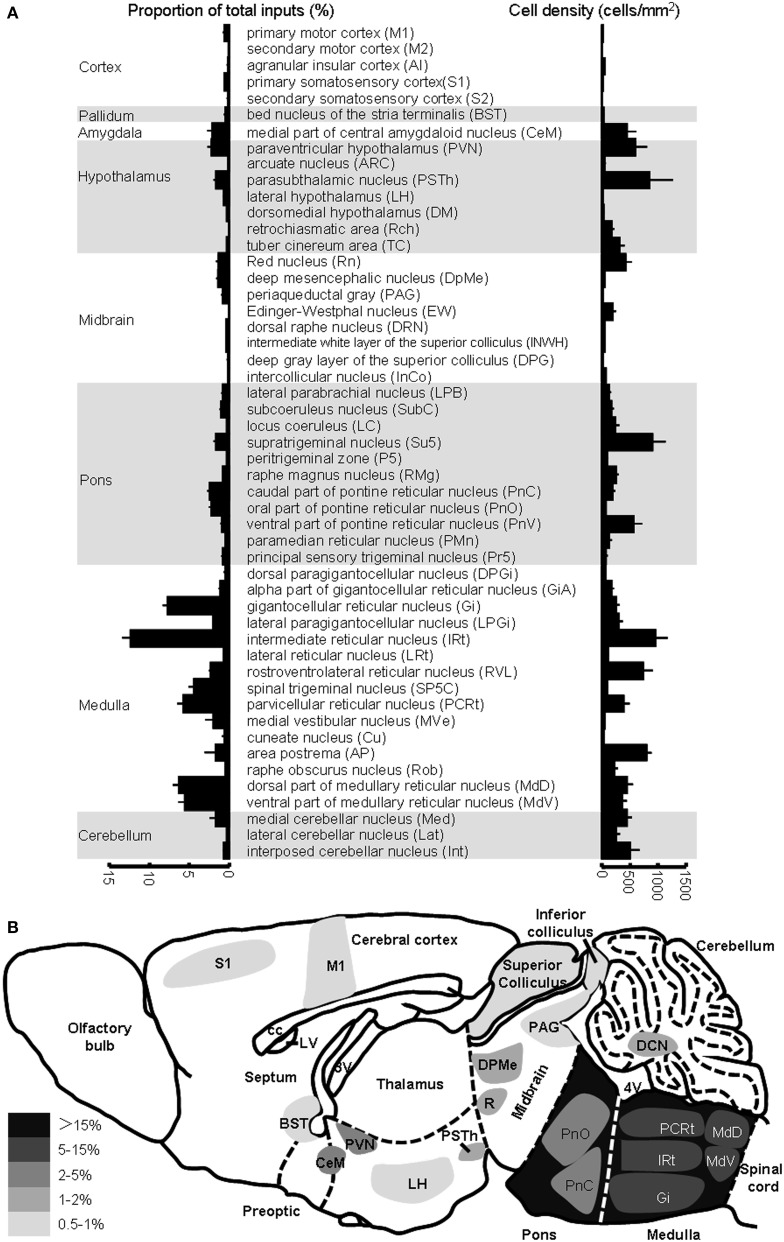
**Brain areas providing inputs to NTS POMC neurons. (A)** Percentage of total input neurons (left) and the density of retrogradely labeled neurons (right) in different brain areas. **(B)** Schematic of major inputs to NTS POMC neurons. DCN, deep nuclei of cerebellum.

Unlike POMC neurons in the ARC, POMC neurons in the NTS predominantly received their inputs from the pons and medulla (~80%) (Figures 6E, 7A). There were also a substantial number of retrogradely labeled neurons in the cerebellum. Those hindbrain inputs were clustered in 29 nuclei, among which the supratrigeminal nucleus (Su5) and IRt were the two most densely labeled areas (Figures 6D, 7A). In the forebrain, ~10% of the total inputs arose from the CeM, PVN, and PSTh (Figures 6C,D, 7A). Some labeling was also sparsely distributed in the ARC, suggesting a rather weak input from the ARC to NTS POMC neurons (Figures 6C, 7A). In the cortex, we found some scattered labeling in the primary motor cortex and somatosensory cortex. No labeling was detected in the vast majority of other cortical areas, hippocampus, striatum, or thalamus. The schematics in Figure 7B illustrate the major brain areas that target NTS POMC neurons.

Although the input patterns for POMC neurons in the ARC and NTS are very different, we did observe several brain areas that served as the common input sources for both groups of POMC neurons. With the exception of the PSTh, the hypothalamic areas that projected to POMC neurons in the NTS also provided direct inputs to POMC neurons in the ARC. Other overlapping areas included the secondary motor cortex (M2), BST, periaqueductal gray (PAG), Edinger-Westphal nucleus (EW), DRN, lateral parabrachial nucleus (LPB), RMg, and raphe obscurus nucleus (ROb). Among the presynaptic partners common to the two POMC neuron populations, BST, DM, LH, retrochiasmatic area (Rch), tuber cinereum area (TC), PVN, ARC, PAG, EW, LPB, RMg, and ROb also provided direct inputs to the AgRP neurons in the ARC (Figures 3, 7). These results suggested that the three groups of projection neurons in the central melanocortin system might be controlled or regulated by some common upstream nuclei.

### Axonal projection patterns of POMC neurons and AgRP neurons

Previous anterograde tracing and immunocytochemistry labeling have revealed that POMC neurons project to the paraventricular thalamic nucleus (PVT) and several hypothalamic nuclei (Fodor et al., 1998; Bagnol et al., 1999; Cowley et al., 2001). In light of the transsynaptic retrograde tracing results that show projections from these areas to POMC neurons, we mapped the axonal projection patterns of the two populations of POMC neurons to test whether these neurons form reciprocal connections with their input nuclei.

**Figure 8:**
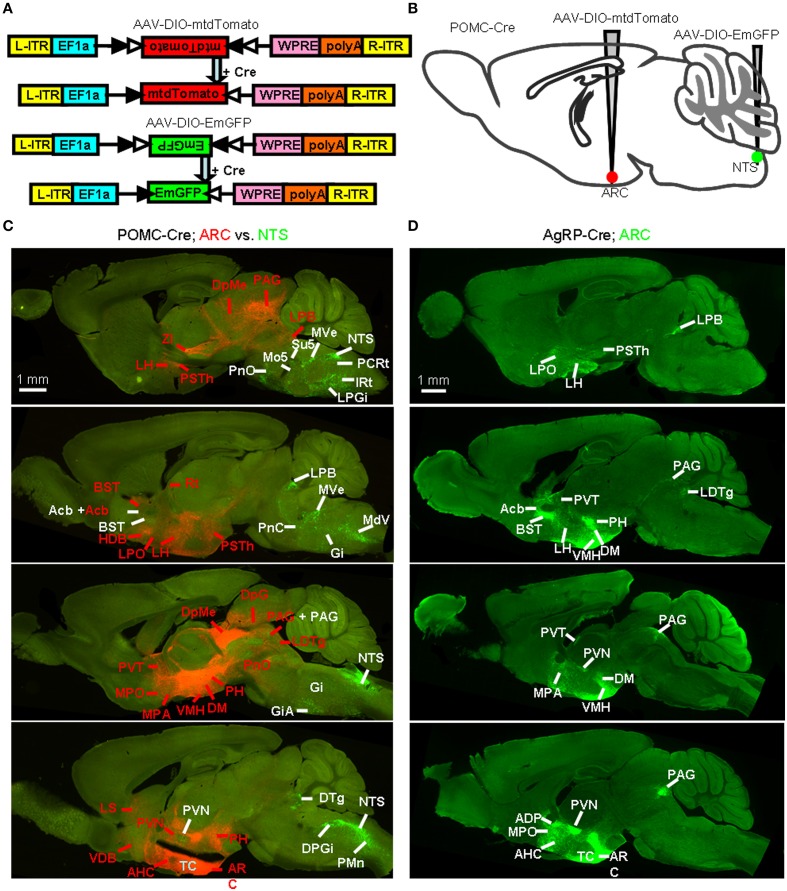
**Identification of whole-brain efferents of POMC neurons. (A)** Design of recombinant AAV strains for Cre-dependent cell labeling. **(B)** AAV-DIO-mtdTomato and AAV-DIO-EmGFP were injected into the ARC and NTS of POMC-Cre mice respectively. **(C)** Direct comparison of the axon projections of the ARC and NTS POMC neurons. The dominant projections of the ARC (red, indicated with red words) and NTS (green, indicated with white words) were represented in a series of sagittal sections. DTg, dorsal tegmental nucleus; Rt, reticular thalamic nucleus. **(D)** Representative sagittal sections show the axon projections of AgRP neurons. LDTg, laterodorsal tegmental nucleus.

We generated two Cre-dependent AAV reporter constructs, AAV-DIO-mtdTomato and AAV-DIO-EmGFP (Figure 8A). These two viral vectors were stereotaxically infused into the ARC and NTS of POMC-Cre mice, respectively (Figure 8B). Following the expression of the two membrane-tagged proteins of mtdTomato or EmGFP, ARC POMC neurons and their axonal fibers exhibited red fluorescence, whereas NTS POMC neurons were green. The green fluorescence of EmGFP was further enhanced using fluorescent immunohistochemistry.

**Figure 9:**
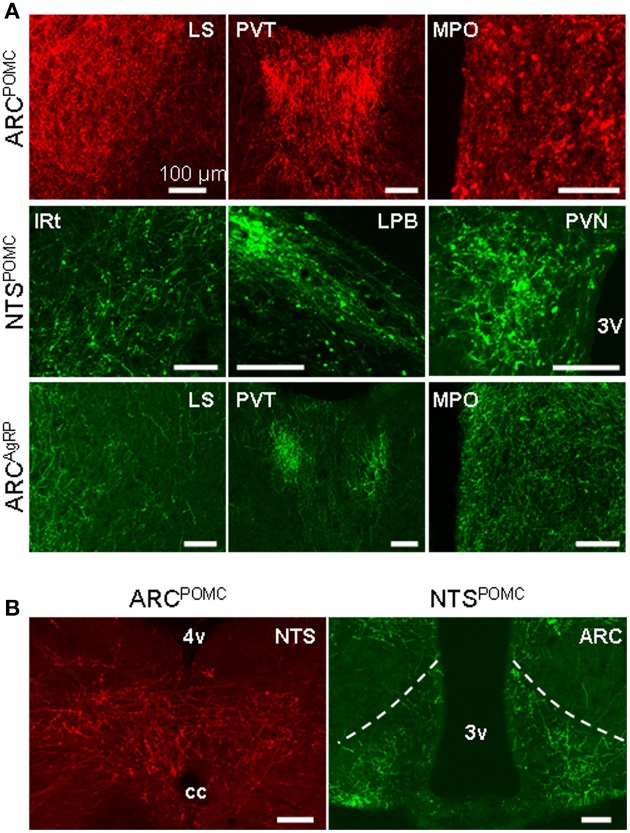
**Brain areas that form reciprocal neurons with POMC and AgRP neurons. (A)** Axon projections from the ARC POMC (upper), NTS POMC (middle) and ARC AgRP (bottom) neurons to their respective input areas. **(B)** ARC POMC neurons project to the NTS (left), and NTS POMC neurons project to the ARC (right). Scale bar: 100 μm.

Figure 8C shows a series of sagittal brain sections to illustrate the overall axon projections patterns of POMC neurons in the ARC and NTS. The heaviest projection of ARC POMC neurons was found in the hypothalamus, especially in the AH, MPA/MPO, LH, DM, VMH, PVN, PSTh, and PH (Figure 8C and Table 1). These axons form numerous branches and varicosities, suggesting that they are true axonal terminals rather than fibers-of-passage (Figure 9A). Elsewhere in the forebrain, dense axonal terminals were observed in the BST, LS, diagonal band of Broca (DB), and accumbens nucleus (Acb). In the midbrain, the PAG, deep gray layer of the superior colliculus (DpG), and deep mesencephalic nucleus (DpMe) received clear innervations from ARC POMC neurons.

**Table 1:** **Brain areas innervated by reciprocal projections from POMC and AgRP neurons**.

**Brain areas**	ARC**^POMC^**	ARC**^AgRP^**	NTS**^POMC^**
LS	^****^	^*^	–
BST	^***^	^****^	–
HDB	^***^	–	–
VDB	^**^	–	–
CeM	–	–	^*^
MPA	^****^	^*^	–
MPO	^****^	^***^	–
LPO	^***^	^***^	–
AH	^***^	^**^	–
PSTh	–	–	^**^
PVN	^***^	^*****^	^**^
LH	^****^	^***^	–
TC	^***^	–	–
SO	^**^	^***^	–
PH	^****^	^***^	–
VMH	^***^	^***^	–
MTu	^***^	^***^	–
ZI	^****^	–	–
PM	^****^	^***^	–
S	^*^	^*^	–
AHi	–	^**^	–
Rn	–	–	^*^
DPMe	–	–	^**^
SuM	^**^	–	–
MM	^***^	–	–
MnR	^***^	–	–
VTg	^****^	–	–
SubC	–	–	^***^
LPB	–	–	^*****^
Su5	–	–	^****^
PnO	–	–	^***^
PnV	–	–	^**^
GiA	–	–	^**^
Gi	–	–	^**^
LPGi	–	–	^*^
RVL	–	–	^*^
SP5C	–	–	^*^
PCRt	–	–	^***^
MdD	–	–	^***^
MdV	–	–	^****^
IRt	–	–	^****^
Med	–	–	^*^

Only those make up greater than 1% of total inputs are listed. Mean fiber intensity (0 ~ 255):

* 1–30;

** 30–60;

*** 60–90;

**** 90–120;

***** *>120; –, null*.

NTS POMC neurons sent their axonal terminals rostrally to as far as the Acb (Figure 8C and Table 1). In the hypothalamus, moderate levels of axonal terminals were found in the PVN and PSTh. Most abundant fibers were found in discrete brainstem areas, including the parvicellular reticular nucleus (PCRt), dorsal and ventral parts of medullary reticular nucleus (MdD and MdV), subcoeruleus nucleus (SubC), Gi, PnO, IRT, Su5, and LPB (Figures 8C, 9A and Table 1).

### Reciprocal projections between POMC/AgRP neurons and their input sources

To systemically analyze the mutual connections between POMC neurons and their presynaptic partners, we examined the axonal density in the brain areas that make up >1% of total inputs to POMC neurons. All presynaptic partners of POMC neurons received reciprocal projections from the POMC neurons (Table 1). For example, ARC POMC neurons projected heavily to all of their major input sources, including the LS, PVT, and MPO (Figure 9A). Similarly, NTS POMC neurons sent reciprocal projections axons to the IRT, LPB, and PVN (Figure 9A). We noted that ARC POMC neurons projected to the NTS, and NTS POMC neurons projected to the ARC (Figure 9B).

As a comparison, the axon projections of AgRP neurons were examined by injecting AAV-DIO-EmGFP into the ARC of AgRP-Cre mice. We observed rather similar projection patterns between POMC and AgRP neurons in the ARC, although the axon projections from POMC neurons were distributed in broader brain areas (Figures 8C,D). In addition, ARC AgRP neurons projected their axonal terminals to all of their dominant input sources, including the LS, PVT, and MPO (Figure 9A and Table 1). Therefore, like ARC POMC neurons, ARC AgRP neurons form reciprocal connections with most of their upstream brain areas.

## Discussion

In this study, we mapped the whole-brain direct inputs to POMC neurons in the ARC and NTS using cell-type specific infection and retrograde spread of modified rabies virus. We found that these two groups of POMC neurons have very different input patterns. POMC neurons in the ARC mainly receive inputs from the hypothalamus and other forebrain nuclei. POMC and AgRP neurons in the ARC share very similar inputs at the level of brain nuclei, although POMC neurons receive broader and denser inputs than AgRP neurons. Unlike ARC POMC neurons, NTS POMC neurons predominantly receive their inputs from the brainstem and cerebellum. Using cell-type specific expression of dual-color fluorescent proteins, we also characterized the axonal projection patterns of POMC neurons in the ARC and NTS. Similar to ARC AgRP neurons, ARC POMC neurons generally target forebrain centers. In contrast, NTS POMC neurons mainly send their axons in the brainstem. Moreover, POMC neurons in the ARC and NTS, as well as AgRP neurons in the ARC, often form reciprocal connections with their respective input centers. These experiments reveal the structural basis underlying the brain regulation of the central melanocortin system and help outline further experiments to dissect the roles of various anatomical pathways in regulating energy homeostasis.

### Comparison between transsynaptic tracing and traditional tracing

Hypothalamic POMC and AgRP neurons have been extensively investigated due to their important roles in feeding behavior and energy homeostasis. However, it had remained unclear whether POMC and AgRP neurons in the ARC are targeted by different brain areas. Previous tract tracing studies have consistently revealed a set of ARC-projecting brain centers, including the PVN, AH, PM, BST, LS, and PAG (Chronwall, 1985; Gruber et al., 1987; Magoul et al., 1993). Using the rabies virus-based approach of transsynaptic retrograde tracing, we found that many of these brain areas provide direct input to both types of ARC neurons. In addition, we detected retrograde labeling in several brain areas, such as the subiculum and zona incerta, that have not been previously identified. This likely reflected the higher sensitivity of the method of transsynaptic tracing using modified rabies virus. Traditional tract tracing studies tended to inject small amount of tracers into part of the ARC to avoid the potential confounding factors of tracer spill-over and tracer pickup by fibers-of-passage. Different research groups often reported variable labeling patterns, particularly for brain areas that provided moderate inputs to the ARC. Thus, both POMC and AgRP neurons in the ARC may integrate inputs from broader brain centers than those revealed using conventional tract tracing methods.

On the other hand, in spite of the increase in the labeling sensitivity, we did not observe any clear transsynaptic labeling in several structures that were identified in traditional tract tracing studies. For example, previous tracing studies reported that there is a projection from the suprachiasmatic nucleus to the ARC (Swanson and Cowan, 1975; Watts et al., 1987), but we did not observe any labeling in this nucleus following rabies virus infection of POMC neurons or AgRP neurons in the ARC. The NTS was reported to project to the ARC in a previous study using horseradish peroxidase (HRP) tracer (Ricardo and Koh, 1978). Consistent with this early finding, we observed the presence of axonal fibers projecting from the POMC neurons in the NTS to the ARC. However, the infection of POMC neurons or AgRP neurons in the ARC did not produce any transsynaptic labeling in the NTS. This difference could be explained by the possibility that neurons in the SCN or NTS target non-AgRP or non-POMC neurons in the ARC. Because of the close proximity between the ARC and SCN, it is also possible that the SCN was non-selectively labeled by the axonal fibers passing through the tracer injection sites in the traditional tracer studies.

### Comparison between POMC neurons and AgRP neurons in the ARC

The overall similarity between the input patterns for POMC neurons and AgRP neurons in the ARC suggests that these two types of neurons integrate and compute signals from largely overlapping brain areas. It is interesting to note that recent transsynaptic tracing studies often have similar input patterns for non-overlapping cell types within the same brain structures, such as the D1- and D2- cells in the striatum (Wall et al., 2013) and serotonergic and GABAergic neurons in the dorsal raphe (Weissbourd et al., 2014). It remains to be tested whether POMC and AgRP neurons in the ARC receive inputs from separate or overlapping populations of input neurons within a given brain structure. Addressing this question requires the development of technique to transsynaptically label neurons with different colors for distinct starter cell populations within the same animal.

In spite of the overall similarity in input patterns, we found that the ARC POMC neurons receive more and broader afferents than AgRP neurons. Moreover, anterograde tracing revealed that ARC POMC neurons send denser and broader outputs than ARC AgRP neurons. In addition to releasing a fast neurotransmitter, such as GABA, POMC neurons may co-release over 10 different hormone products, including ACTH, beta-endorphin, and melanocyte-stimulating peptides (Millington, 2007). Therefore, POMC neurons integrate a wide array of brain inputs and modulate a broad set of downstream neurons using various signaling molecules.

### Comparison between POMC neurons in the ARC and the NTS

POMC neurons in the ARC and NTS respond to different signals from the brain and periphery and suppress feeding behaviors at different time scales (Cowley et al., 2001; Fan et al., 2004; Appleyard et al., 2005; Vong et al., 2011; Berglund et al., 2012; Zhan et al., 2013). Unlike their counterparts in the ARC, POMC neurons in the NTS mainly integrate inputs from the brainstem and provide outputs to brainstem structures. Many of these structures play essential roles in controlling and regulating consummatory ingestive behaviors. For example, IRt, PCRt, and Rn are implicated in chewing and swallowing (Chen et al., 2001; Satoh et al., 2008; Travers et al., 2010; Stanek et al., 2014). The difference in anatomical connections likely underlies the functional specialization for POMC neurons in the hypothalamus and brainstem. On the other hand, our tracings have identified 12 nuclei that provide direct inputs to the NTS POMC neurons, ARC POMC neurons, and ARC AgRP neurons. These structures include the PVN, the major center for regulating energy homeostasis (Leibowitz et al., 1981; Stanley and Leibowitz, 1985), and the RMg and Rob, two brainstem centers of serotonergic neurons (Bowker et al., 1981, 1983; Azmitia and Gannon, 1986). These common inputs may provide anatomical substrates for the concerted regulation of feeding behavior by targeting all three types of projection neurons in the melanocortin system.

### Functional implications

Our detailed mapping of the connectivity points to functional studies on the roles and circuit mechanisms of POMC neurons. Recent studies have deciphered the functions of several neural pathways to AgRP neurons (Krashes et al., 2014). Although numerous studies suggest the connections from the PVN to ARC POMC neurons and the importance of the PVN in the feeding behavior (Leibowitz et al., 1981; Stanley and Leibowitz, 1985; Cowley et al., 1999; Balthasar et al., 2005; Atasoy et al., 2012), relatively little is known about the electrophysiological effects and functions of the projections from the PVN to POMC neurons in the ARC and NTS.

The lateral septum is another subcortical area that provides inputs to both POMC neurons and AgRP neurons in the ARC. Neuronal activity in the lateral septum is affected by gastric distension and ghrelin administration (Gong et al., 2013). Moreover, administering either opioid or noradrenaline into the septal nuclei increases food intake in rats (Majeed et al., 1986; Scopinho et al., 2008). It remains to be tested how the inputs from lateral septal neurons affect the physiology of ARC POMC and AgRP neurons as well as regulate feeding behavior.

In addition to the inputs from upstream stations to POMC and AgRP neurons, our anterograde tracing also reveals extensive projections from these neurons back to a majority of their input sources. The PVN, in particular, forms reciprocal connections with all three populations of melanocortin projection neurons. Optogenetic studies have demonstrated that the reciprocal projections between ARC AgRP neurons and PVN neurons promote feeding (Betley et al., 2013; Krashes et al., 2014). However, the functions of the vast majority of reciprocal projections have not yet been explored. Such functional studies will help elucidate the computational mechanisms underlying the regulation of energy homoeostasis.

ARC POMC neurons consist of a heterogeneous subpopulation of distinct neurotransmitter phenotypes (Hentges et al., 2004, 2009; Meister et al., 2006). Given this heterogeneity and the functional diversity of POMC neurons (Sohn and Williams, 2012), it is possible that different subsets of POMC neurons may be linked to specific subcircuits and mediate distinct physiological and behavioral functions. A recent study shows that distinct subpopulations of ARC AgRP neurons target different brain regions and not all of these neuronal subpopulations are sufficient to evoke feeding (Betley et al., 2013). It will be valuable to more precisely dissect neural circuits at the level of subpopulations of POMC and AgRP neurons.

## Author contributions

ML, FX and CZ designed the research; XH prepared the rabies virus; QF prepared the AAV; DW and ZZ performed the tracing experiments and analyzed data; YS performed the 3D reconstructions; ML and CZ wrote the paper.

### Conflict of interest statement

The authors declare that the research was conducted in the absence of any commercial or financial relationships that could be construed as a potential conflict of interest.

## Supplementary material

Supplementary Image 1 **3D visualization of whole-brain direct inputs to the ARC POMC neurons**.

Supplementary Image 2 **3D visualization of whole-brain direct inputs to the ARC AgRP neurons**.

Supplementary Image 3 **3D visualization of whole-brain direct inputs to the NTS POMC neurons**.

## Abbreviations

Acb: accumbens nucleus
ADP: anterodorsal preoptic nucleus
AH: anterior hypothalamus
AHi: amygdalohippocampal area
AI: agranular insular cortex
AP: area postrema
ARC: arcuate nucleus
ATg: anterior tegmental nucleus
BST: bed nucleus of the stria terminalis
CeM: medial part of the central amygdaloid nucleus
Cg: cingulate cortex
Cu: cuneate nucleus
DB: diagonal band of Broca
DCN: deep nuclei of cerebellum
DM: dorsomedial hypothalamus
DP: dorsal peduncular cortex
DpG: deep gray layer of the superior Colliculus
DPGi: dorsal paragigantocellular nucleus
DpMe: deep mesencephalic nucleus
DRN: dorsal raphe nucleus
DS: dorsal subiculum
DTg: dorsal tegmental nucleus
DTT: dorsal tenia tecta
EW: Edinger-Westphal nucleus
Gi: gigantocellular reticular nucleus
GiA: alpha part of gigantocellular reticular nucleus
HDB: horizontal diagonal band of broca
IL: infralimbic cortex
InCo: intercollicular nucleus
INWH: intermediate white layer of the superior Colliculus
Int: interposed cerebellar nucleus
IRt: intermediate reticular nucleus
LA: lateroanterior hypothalamic nucleus
Lat: lateral cerebellar nucleus
LC: locus coeruleus
LDT: glaterodorsal tegmental nucleus
LH: lateral hypothalamus
LHb: lateral habenular nucleus
LPB: lateral parabrachial nucleus
LPGi: lateral paragigantocellular nucleus
LPO: lateral preoptic area
LRt: lateral reticular nucleus
LS: lateral septum
M1: primary motor cortex
M2: secondary motor cortex
MdD: dorsal parts of medullary reticular nucleus
MdV: ventral parts of medullary reticular nucleus
MEA: medial amygdaloid nucleus
Med: medial cerebellar nucleus
MM: medial mammillary nucleus
MnR: median raphe nucleus
MO: medial orbital cortex
MPA: medial preoptic area
MPO: medial preoptic nucleus
MS: medial septum
MTu: medial tuberal nucleus
MVe: medial vestibular nucleus
NI: nucleus incertus
NTS: nucleus tractus solitaries
P5: peritrigeminal zone
PAG: periaqueductal gray
PCRt: parvicellular reticular nucleus
PH: posterior hypothalamus
PM: premammillary nucleus
PMn: paramedian reticular nucleus
PnC: caudal parts of the pontine reticular nucleus
PnO: oral parts of the pontine reticular nucleus
PnV: ventral part of pontine reticular nucleus
Pr5: principal sensory trigeminal nucleus
PrL: prelimbic cortex
PSTh: parasubthalamic nucleus
PVN: paraventricular hypothalamic nucleus
PVT: paraventricular thalamic nucleus
Rch: retrochiasmatic area
RMg: raphe magnus nucleus
Rn: red nucleus
Rob: raphe obscurus nucleus
RS: retrosplenial cortex
RSG: retrosplenial granular cortex
Rt: reticular thalamic nucleus
RVL: rostroventrolateral reticular nucleus
S1: primary somatosensory cortex
S2: secondary somatosensory cortex
S: subiculum
SCN: suprachiasmatic nucleus
SLEA: sublenticular extended amygdala
SO: supraoptic nucleus
SP5C: spinal trigeminal nucleus
Su5: supratrigeminal nucleus
SubC: subcoeruleus nucleus
SuM: supramammillary nucleus
TC: tuber cinereum area
VMH: ventromedial hypothalamic nucleus
VP: ventral pallidum
VS: ventral subiculum
VTA: ventral tegmental area
VTg: ventral tegmental nucleus
ZI: zona incerta
3V: third ventricle
4V: fourth ventricle
cc: central canal
LV: lateral ventricle
AgRP: Agouti-related peptide
POMC: Pro-opiomelanocortin.
